# The Effect of Low Temperature on Laying Performance and Physiological Stress Responses in Laying Hens

**DOI:** 10.3390/ani13243824

**Published:** 2023-12-11

**Authors:** Da-Hye Kim, Ju-Yong Song, Jina Park, Byung-Yeon Kwon, Kyung-Woo Lee

**Affiliations:** Department of Animal Science and Technology, Konkuk University, 120 Neungdong-ro, Gwangjin-gu, Seoul 05029, Republic of Korea; kdh142536@naver.com (D.-H.K.); 0jysong970@gmail.com (J.-Y.S.); jinaa97@konkuk.ac.kr (J.P.); byung64@konkuk.ac.kr (B.-Y.K.)

**Keywords:** cold stress, egg production, egg quality, lipid metabolism, body temperature, laying hens

## Abstract

**Simple Summary:**

Cold stress is considered an environmental stress and is an important managemental factor in regions where winter temperatures drop below 18 °C. Although a low temperature lowered the productive performance of laying hens, there are still unanswered questions as to the physiological changes that occur when they are exposed to low temperature, especially changes in antioxidants and stress indicators, which prompted us to set up the current study. Laying hens reared at low temperature (12 ± 4.5 °C) showed impaired laying performance compared with laying hens reared at normal temperature (24 ± 3 °C). However, eggshell color was intensified in laying hens exposed to low temperature. During the early stage of exposure to low-temperature stress, malondialdehyde levels in the egg yolk increased in laying hens raised at low temperature vs. normal temperature. However, yolk corticosterone, an indicator of stress responses, remained unchanged. Low temperature had an impact on total cholesterol and triglyceride levels in serum increases in laying hens reared at low temperature. In essence, low temperature in laying hens altered antioxidant systems and lipid metabolism without inducing stress responses.

**Abstract:**

The present study investigated the effect of low temperature on laying performance, egg quality, body temperature, yolk malondialdehyde, yolk corticosterone, and serum biochemistry in laying hens. A total of 40 laying hens (Hy-Line Brown) aged 36 weeks were housed in one of two environmental chambers kept at 12 ± 4.5 °C (low temperature) or 24 ± 3 °C (normal temperature) for 4 weeks. Low vs. normal temperature significantly increased (*p* < 0.05) live body weight, feed intake, and feed conversion ratio in laying hens. Skin surface temperature, but not rectal temperature, was decreased in laying hens exposed to low vs. normal temperature. Hens exposed to low temperature laid an intense eggshell color compared with those raised in a normal temperature. Malondialdehyde concentrations in yolk were increased in low-temperature-exposed laying hens compared with those at normal temperature conditions, but this effect was only noted on day 7, post the low-temperature exposure (*p* = 0.04). Finally, low vs. normal temperature increased the concentrations of total cholesterol and triglyceride in serum. Collectively, this study indicates that exposure to low temperature in laying hens initially disrupted antioxidant system and altered lipid metabolism in laying hens without inducing stress responses.

## 1. Introduction

The future of poultry husbandry will be focused on improved animal welfare, with less reliance on preventative medical interventions. In the poultry husbandry environment, animals are likely to encounter various kinds of simultaneous environmental stressors. In emphasizing the latter, authors have tried to understand the physiological and stress responses of laying hens exposed to various stressors, including stocking density, transport, or ambient temperature [1]. It is generally understood that the thermal neutral zone for the optimal metabolic and productive activity of poultry ranges from around 18 to 23.9 °C [2] with a relative humidity between 50% and 70% during the laying period [3]. Heat stress is well-explored and acknowledged when laying hens are raised beyond the thermal neutral zone [1]. In contrast to heat stress, our understanding of the impact of low temperature (i.e., cold stress) on laying hens is still limited. 

It has been reported that the ambient environmental temperature below 16 °C results in negative effects on poultry production performance, such as egg production, egg mass, and egg quality [4,5,6]. Cold stress is considered an environmental stress and can be considered an important managemental factor in regions where environmental temperatures during the winter periods drop below 18 °C. Earlier studies reported that cold exposure could influence the antioxidant and immune system functions of the host [7,8,9]. During low-temperature exposure, the hypothalamus, which acts as the control center for temperature regulation, triggers homeostatic mechanisms to maintain body temperature by generating heat. Also, upon experiencing stress, the activation of the hypothalamic–pituitary–adrenal (HPA) axis leads to the elevated secretion of corticosterone (CORT) from the adrenal glands. This process is intended to stimulate gluconeogenesis within various tissues and lipolysis in adipose tissue, thereby augmenting both glucose accessibility and the metabolic rate of energy [10]. 

In general, low temperature, as a physical environmental stressor, is known to in-crease feed intake to meet energy requirements [11]. Low ambient temperatures also result in decreased insulin and increased corticosterone (CORT) concentrations in the serum of laying hens [12,13]. It is well-known that insulin and CORT are influenced by temperature and these hormones play an important role in controlling nutrient metabolism [14,15]. In addition, a low temperature can disrupt the balance in an antioxidant system, leading to oxidative damage of several tissues by altering the enzymatic and non-enzymatic antioxidant status, lipid peroxidation, antioxidant vitamins, and reactive oxygen species production [16]. It has been reported that malondialdehyde (MDA) was increased in chickens exposed to low temperature, when compared to normal-temperature-reared counterparts [17,18]. Nonetheless, studies of the effects of low temperature on the egg quality, body temperature (rectal and surface), MDA in yolk, and CORT in yolk of laying hens that engage on a weekly basis are scarce, which prompted us to set up the current experiment. Thus, this present study aimed to address the biological and physiological responses of laying hens exposed to low ambient temperature.

## 2. Materials and Methods

### 2.1. Birds, Diets, and Experimental Design

A total of forty 36-week-old Hy-Line Brown laying hens with the average body weight of 1871 ± 123 g were used in this study. The birds were housed (one bird/cage) in one of two experimental chambers, which had one tier of 20 cages, 1 m high from the floor. Each cage, measuring 41 × 37 × 40 cm (length × depth × height), had nipples and a trough-type feeder. Two adjacent cages sharing one feeder were considered a replicate (n = 2/replicate, 10 replicates/treatment). The sample size (n = 10 per treatment) was calculated based on a Type I error of 5%, 80% statistical power, an effect size of 7.6%, and a coefficient of variation of 6%. In addition, to reduce variation within replicates, 2 laying hens per replicate were used. Each hen was provided with a 1517 cm^2^ floor space. All birds were initially adapted to the chambers for 7 days at an ambient temperature of 24 °C with a relative humidity of 40 ± 4% and a lighting program of 16L/8D. After adaptation periods, hens were exposed to one of two temperature regimes (see Section 2.2 for the temperature regimes) for 28 days. Corn, soybean meal, and dried distillers grains with a solubles-based commercial layer diet were provided (Table 1), with feed and water supplied ad libitum.

### 2.2. Temperature Treatments and Temperature Monitor

Two environmental chambers were continuously set at 12 ± 4.5 °C (i.e., low temperature) and 24 ± 3.5 °C (i.e., normal temperature) and the average relative humidity inside the chambers was maintained at 40 ± 4%. Each chamber was equipped with a heater (MCP-300; MAXCON Co., Bucheon, Republic of Korea), air-conditioner (AR07J5174HA, SAMSUNG, Suwon, Republic of Korea), humidifier (MH-601A; mtechwin Co., Gimhae, Republic of Korea), dehumidifier (NED-062P; NAWOOEL Co., Gimpo, Republic of Korea), and the main controller panel. Temperature and humidity loggers (MHT-381SD; Lutron Electronic Enterprise Co., Taipei, Taiwan) were also installed to monitor the temperature and relative humidity at 10 min intervals throughout the experiment.

### 2.3. Measurements of Rectal Temperature and Skin Surface Temperature

On days 7, 14, 21, and 28 following temperature treatment, the rectal and skin surface temperature of laying hens was measured. One hen per replicate was randomly selected and measured by inserting a rectal thermometer to a depth of 3 cm into the rectum. Skin surface temperature was measured at three different sites (i.e., head, chest, and leg) using a thermal imaging camera (Cat^®^ S60: equipped with an FLIR™ Lepton, FLIR Systems Inc., Wilsonville, OR, USA), as previously described [19]. The hens were handled by wearing latex gloves, to avoid the influences of heat and moisture of the hands on the temperature of the feathers.

### 2.4. Egg Production and Egg Quality

Body weight and feed intake were measured weekly. Eggs were collected daily and weighed per replicate to calculate hen-day egg production. Feed conversion ratio was expressed as kg of feed consumed per kg of eggs produced. Eggs (3 eggs per replicate) collected on the preceding 3 consecutive days, at 7, 14, 21, and 28 days following the temperature treatment, were used to measure egg quality with a digital egg tester (DET-6000, Navel, Kyoto, Japan). Yolk color intensity was evaluated on a scale between 1 and 16, with 1 being a very pale yellow and 16 being a dark orange. Eggshell color was measured using an egg multi-tester made by TSS (QCR, Technical Services and Supplies Ltd., Yolk, UK). Eggshell color intensity was evaluated on a scale between 0 and 100, with 0 being a darkness and 100 being a lightness.

### 2.5. Malondialdehyde in Yolk Samples

Eggs were collected to measure yolk MDA concentrations at days 7, 14, 21, and 28. The eggs were cracked open, and the yolks were separated from the albumin by gently rolling the yolk on filter paper. Three yolks were pooled and homogenized. The yolk MDA was measured using the OxiSelect TBARS Assay kit (Cell Biolabs Inc., San Diego, CA, USA).

### 2.6. Corticosterone in Yolk Samples

Yolk CORT concentrations were measured at 7, 14, 21, and 28 days, and 4 g of pooled yolk were vortexed with an equal volume of PBS. Then, 1 mL of the yolk suspension was mixed with an equal volume of ethanol, incubated at 37 °C for 1 h, and subsequently centrifuged. The 50 μL of supernatants were mixed with 50 μL of ethanol and 50 μL of PBS solution, and these mixtures were analyzed with a CORT ELISA kit (Enzo life science Inc., ADI-901-097, Farmingdale, NY, USA), as previously described [20,21].

### 2.7. Serum Parameters

At 28 days, one bird per replicate was selected to collect blood from a wing vein. Serum was separated by centrifugation at 200 g for 15 min and stored at −20 °C until the analysis. Serum samples were analyzed by using an automatic blood chemical analyzer (Film DRI CHEM 7000i, Fuji film, Tokyo, Japan) to measure for total cholesterol, triglyceride, high-density lipoprotein cholesterol, glutamic oxaloacetic transaminase, glutamic pyruvic transaminase, and uric acid.

### 2.8. Statistical Analysis

Two adjacent cages were considered an experimental unit. All data were analyzed using Student’s *t*-test procedure of SAS (SAS Institute Inc., Cary, NC, USA). Results were presented as least square means and standard deviation. Differences were considered significant at *p* < 0.05.

## 3. Results

**Figure 1:**
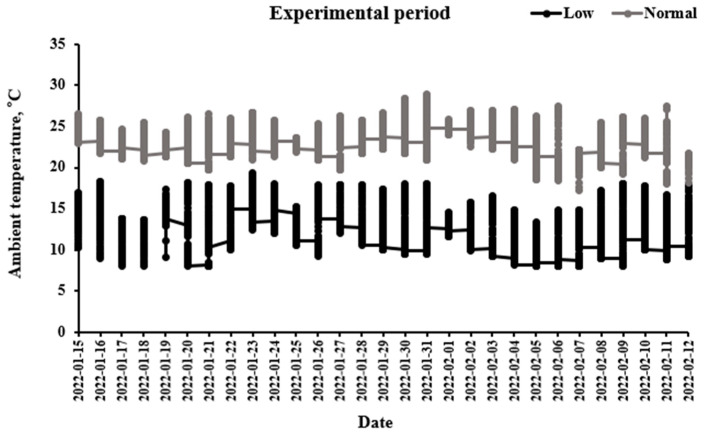
Ambient temperatures in chambers were recorded at an interval of 10 min with a thermometer.

Ambient temperature ranged from 21 to 27 °C (normal temperature) and from 7.5 to 16.5 °C (low temperature) in the normal- and low-temperature chambers (Figure 1). 

Low temperature increased (*p* < 0.05) the final body weight, feed intake, and FCR of laying hens compared with those raised at a normal temperature (Table 2). 

However, egg weight, egg production and egg mass were not affected (*p* > 0.05) by temperature regimes. Rectal temperature during the experimental period was maintained from 41.27 to 41.58 °C and was not different between temperature treatment groups (Table 3). 

Table 4 presents the skin surface temperature at 7, 14, 21, and 28 days. The laying hens exposed to low temperature at all ages had lower skin (i.e., head, breast, and leg regions) surface temperature compared with those raised at normal temperature (*p* < 0.05). Skin surface temperature between low- vs. normal-temperature treatments was 5.7 to 12.2 °C lower in the breast surface area, followed by the legs and the head areas. 

Low temperature significantly increased (*p* < 0.05) eggshell color at days 7 and 28, compared with normal-temperature-raised laying hens (Table 5). However, yolk color, the Haugh unit, shell strength, and shell thickness were not affected by temperature treatments on all days (*p* > 0.05). 

Yolk MDA concentrations were higher in laying hens exposed to low temperature, compared with those in normal temperature at 7 days (Table 6). However, low-temperature-induced decreases in yolk MDA concentrations were not noted (*p* > 0.05) at 14, 21, and 28 days. 

The CORT concentrations were not different in laying hens exposed to low and normal temperature (Table 7). Low vs. normal temperature increased (*p* < 0.05) the concentrations of total cholesterol and triglycerides, but did not affect the concentrations of high-density lipoprotein cholesterol, glutamic oxaloacetic transaminase, glutamic pyruvic transaminase, and uric acid in serum samples (Table 8; *p* > 0.05).

## 4. Discussion

The present study showed that low vs. normal ambient temperature increased final body weight, feed intake, and FCR in laying hens. This finding is well-established because chickens, as a homeothermic animal, are unable to warm themselves and so overfeed to compensate for heat loss to the environment when they are exposed to low temperature (e.g., 18 °C) [22]. As ambient temperature shifted from 30 to 18 °C, the abdominal fat weight, abdominal fat rate, and subcutaneous fat thickness linearly increased, favoring body fat deposition [23]. For these reasons, the present study indicates that heavier body weight is due to increases in feed intake and concomitant body fat deposition, leading to a higher FCR in laying hens exposed to low vs. normal temperature. It is well-documented that low temperatures stimulate feed intakes in laying hens [6,24], broiler chickens [17,25], quails [4,26] and turkeys [27].

In the present study, rectal temperature at all ages was not altered in birds exposed to a low temperature or those at normal temperature, which agrees with previous studies with turkeys [28] and laying hens [3]. These results suggest that body heat exchange (e.g., production and loss) does not change above or below the normal rectal temperature range when the homeostasis of warm-blooded animals is maintained [29]. On the other hand, skin surface temperature, an important evaluable parameter, quickly shifts in response to environmental changes and serves as an indicator of alterations in peripheral blood flow and heat exchange [30]. Skin surface (e.g., head, chest, and leg) temperatures were significantly decreased in laying hens raised at low temperature, compared with those at normal temperature, during the whole experiment period. In line with our finding, lower skin surface temperatures in broiler chickens [31] and laying hens [29] have been observed when they were exposed to low vs. normal ambient temperature. It is understood as a way of preventing the loss of heat by cold-induced peripheral vasoconstriction, which reduces blood circulation to the body surface [31]. Additionally, the temperatures of the head and legs were kept higher than that of the chest. It is speculated that this is because the head and legs are featherless body surfaces, compared with the breast body surface, which is covered by feathers [30]. 

We found that yolk color and eggshell strength were not altered in laying hens raised at a low temperature compared with normal-temperature-raised laying hens, although the former ate more. In contrast to our belief [24,32], our finding indicates that hens exposed to low temperatures might not use excess feed-origin carotenoids and essential nutrients (e.g., amino acids, Ca, Mg) to intensify yolk and to increase eggshell strength. It is of interest that low-temperature-raised hens laid more intensified eggshell colors than hens raised in normal ambient temperature. It is known that the endogenously synthesized protoporphyrin IX is the major pigment in brown eggshell, and that stress factors, including stocking density, fear, or molting, often deteriorate eggshell color pigmentation in brown laying hens [33]. As the pigment is known to be synthesized in the shell gland of the oviduct, it seems that low temperature per se may stimulate the synthetic process of pigment or effectively deposit it on the shell layers. Further studies are warranted to reveal the underlying mechanisms that will show how low temperature affects shell color deposition in laying hens. 

It is reported that cold stress causes tissue damage [22] via increased metabolic rate, which demands tissue requirement for oxygen in birds [34]. We found that yolk MDA levels were elevated in low- vs. normal-temperature treatments, but this effect was only noted at 7 days post temperature exposure. Rahmani et al. [17] reported that broiler chickens exposed to 15 °C vs. 22 °C had increased plasma MDA concentrations. In addition, MDA levels in plasma and liver samples were significantly higher in broiler chickens in the low temperature group (from 10 to 15 °C) at 21 and 42 days [18]. 

In contrast to yolk MDA alterations caused by low temperature, no differences in yolk CORT were noted between low-temperature and normal-condition groups. Exposure of chickens to stress increases the secretion of CORT, a major stress hormone in chickens, via the activation of the hypothalamic pituitary adrenal axis [35]. In earlier studies, the levels of CORT in the albumen and yolk of chicken eggs were used as a non-invasive method to measure stress [20,36,37]. The substances or hormone concentrations in the blood during the egg’s formation phase are deposited in the yolk composition. Hence, if there is an increase in plasma CORT, it would consequently be transferred into the eggs [36]. Also, catching animals for blood sampling can be stressful and it also causes an increase in CORT [38]–therefore, egg yolks were used for an analysis of CORT concentrations in this study. Conflicted results have been reported regarding plasma CORT in response to low ambient temperature. With various treatments (durations of cold exposure, temperature, and age), increased plasma levels of CORT were reported in young broiler chickens (19  ±  1 °C, 6 h/day, from the first to the seventh day of life) [39], male turkeys [40], chicks (1.2 °C for 3 h) [41], and laying hens (0 °C for 72 h) [42]. On the other hand, Hangalapura et al. [43] reported that low temperature (10.4 ± 0.5 °C) decreased plasma CORT in laying hens and Hu and Cheng [44] reported no effect of low temperature on the CORT level in laying hens. It is not clear at this stage why low temperature failed to affect yolk CORT levels in this study, although the information on the effect of low temperature in yolk CORT is scarce. The unaltered CORT could be due to the negative feedback of CORT on the hypothalamus axis with chronic low-temperature exposure, resulting in an inhibition of adrenocorticotropin secretion [45]. Similarly, previous studies have reported that consistently increased CORT is not commonly seen during chronic stress, possibly due to the negative feedback of CORT on the hypothalamus axis [43,44,46]. 

The finding that low temperature increased the concentrations of total cholesterol and triglyceride is in agreement with the results of [17,47]. This finding indicates that a low-temperature-induced increase in feed intake, coupled with cold stress response in chickens, could stimulate hepatic lipogenic and hypercholesterolemic metabolic pathways [48,49]. On the other hand, glutamic oxaloacetic transaminase and glutamic pyruvic transaminase were not altered by low temperatures, indicating the maintenance of the hepatic function.

## 5. Conclusions

In conclusion, low vs. normal ambient temperature stimulated feed intake and in-creased body weight and FCR. Eggshell color was intensified in laying hens exposed to low temperature. Low temperature elevated skin surface temperature without affecting rectal temperature. Finally, low temperature did not influence stress responses, as manifested by constant yolk CORT concentrations, but altered MDA, total cholesterol, and triglyceride levels. Taken together, our study indicates that the exposure of laying hens to low temperature disrupted the antioxidant system, especially at an early stage of exposure, and altered lipid metabolism (i.e., total cholesterol and triglyceride) without inducing stress responses. The low-temperature-mediated increase in eggshell color seen in this study warrants further study.

## Figures and Tables

**Table 1:** Ingredients and chemical composition of the basal diet (%, as-fed basis).

Ingredients	g/100 g
Corn	55.122
Corn dried distillers grains with solubles	13.500
Soybean meal, 44% CP	5.000
Wheat, 12% CP	4.000
Corn gluten meal, 60% CP	1.861
Rapeseed meal	1.933
Sesame meal	1.800
Meat meal	3.000
Feather meal	1.000
Full-fat soybeans	0.900
L-lysine-HCl, 78%	0.242
DL-methionine, 98%	0.152
Monocalcium phosphate	0.458
L-Threonine	0.025
Choline, 50%	0.200
Salt	0.230
Limestone	10.387
Vitamin premix ^1^	0.120
Mineral premix ^2^	0.070
Total	100.00
Calculated values	
AMEn, kcal/kg	2800
Crude protein	18.02
Crude fat	3.82
Ca	3.95
Total phosphorus	0.64
Methionine + Cysteine	0.80
Lysine	0.84
Threonine	0.63
Tryptophan	0.20

^1^ Vitamin premix provided following nutrients per kg of diet: vitamin A, 24,000 IU; vitamin D3, 5520 IU; vitamin E, 48 mg; vitamin K3, 7.80 mg; thiamine, 4.32 mg; riboflavin, 9.60 mg; vitamin B6, 6.96 mg; vitamin B12, 0.05 mg; niacin, 72 mg; biotin, 0.30 mg; pantothenic acid, 22.04 mg; folate, 1.68 mg. ^2^ Mineral premix provided following nutrients per kg of diet: Fe, 49 mg; Mn, 56 mg; Zn, 42 mg; I, 0.7 mg; Se, 0.14 mg; Cu, 5.25 mg; Co, 0.09 mg.

**Table 2:** The effect of cold stress on production performance ^1^.

Item	Temperature				*p*-Value
	Low ^3^		Normal		
	Mean	SD ^4^	Mean	SD	
0 to 28 days					
Initial body weight, kg	1.867	0.128	1.923	0.107	0.302
Final body weight, kg	2.073	0.063	1.993	0.073	0.018
Feed intake, g	133.7	5.273	112.7	8.655	<0.001
FCR ^2^, kg/kg	2.691	0.451	2.020	0.132	<0.001
Egg weight, g	64.04	1.861	65.28	2.702	0.248
Egg production, %	89.82	8.164	92.68	4.795	0.353
Egg mass	55.52	5.236	57.73	3.023	0.263

^1^ *n* = 10 replicates per treatment. ^2^ FCR = feed conversion ratio (kg of feed consumed per kg of eggs produced). ^3^ Low = low temperature (12 ± 4.5 °C); Normal = normal temperature (24 ± 3 °C). ^4^ SD= standard deviation.

**Table 3:** The effect of cold stress on rectal temperature (°C) ^1^.

Item	Temperature				*p*-Value
	Low ^2^		Normal		
	Mean	SD ^3^	Mean	SD	
Day 7	41.58	0.175	41.52	0.248	0.507
Day 14	41.44	0.193	41.41	0.242	0.802
Day 21	41.41	0.280	41.27	0.342	0.330
Day 28	41.28	0.190	41.40	0.148	0.133

^1^ *n* = 10 replicates per treatment. ^2^ Low = low temperature (12 ± 4.5 °C); Normal = normal temperature (24 ± 3 °C). ^3^ SD = standard deviation.

**Table 4:** The effect of cold stress on skin surface temperature (°C) ^1^.

Item	Temperature				*p*-Value
	Low ^2^		Normal		
	Mean	SD ^3^	Mean	SD	
Day 7					
Head	36.31	0.693	38.49	0.994	<0.0001
Breast	21.04	0.918	29.10	0.460	<0.0001
Leg	34.89	1.343	39.10	0.330	<0.0001
Day 14					
Head	37.93	0.380	39.75	0.483	<0.0001
Breast	25.39	1.514	31.09	0.437	<0.0001
Leg	38.27	0.853	40.49	0.461	<0.0001
Day 21					
Head	36.26	0.421	39.97	0.447	<0.0001
Breast	21.20	0.511	32.19	0.746	<0.0001
Leg	35.47	1.309	40.72	0.472	<0.0001
Day 28					
Head	36.92	0.684	40.27	0.330	<0.0001
Breast	20.13	0.528	32.29	0.330	<0.0001
Leg	36.16	0.986	41.11	0.372	<0.0001

^1^ *n* = 10 replicates per treatment. ^2^ Low = low temperature (12 ± 4.5 °C); Normal = normal temperature (24 ± 3 °C). ^3^ SD = standard deviation.

**Table 5:** The effect of cold stress on egg quality ^1^.

Item	Temperature				*p*-Value
	Low ^4^		Normal		
	Mean	SD ^5^	Mean	SD	
Yolk color ^2^					
Day 7	8.490	0.239	8.457	0.344	0.521
Day 14	8.486	0.243	8.652	0.528	0.182
Day 21	8.364	0.190	8.479	0.311	0.582
Day 28	8.326	0.325	8.236	0.310	0.252
Haugh unit					
Day 7	90.98	1.924	91.32	2.789	0.807
Day 14	93.43	2.265	94.38	2.999	0.379
Day 21	95.81	1.479	93.83	5.683	0.333
Day 28	92.70	2.616	93.93	2.590	0.535
Shell strength, kgf					
Day 7	5.126	0.323	5.319	0.802	0.748
Day 14	5.247	0.493	5.248	0.855	0.436
Day 21	5.500	0.404	4.984	0.666	0.333
Day 28	5.285	0.459	5.285	0.453	0.306
Shell thickness, mm					
Day 7	0.399	0.024	0.434	0.029	0.490
Day 14	0.410	0.023	0.398	0.026	0.998
Day 21	0.437	0.020	0.424	0.021	0.051
Day 28	0.403	0.024	0.436	0.024	0.997
Eggshell color ^3^					
Day 7	26.97	2.156	25.83	3.395	0.010
Day 14	27.26	3.041	25.77	3.176	0.295
Day 21	27.83	3.567	25.13	2.161	0.170
Day 28	27.76	2.806	24.71	2.282	0.007

^1^ *n* = 10 replicates per treatment. ^2^ Yolk color was determined using a digital egg tester (DET-6000, Navel, Kyoto, Japan); scale between 1 to 16, with 1 being a very pale yellow and 16 being a dark orange. ^3^ Eggshell color was measured by an Egg multi tester made by TSS (Technical Services and Sup-plies Ltd., Yolk, UK); scale between 0 to 100, with 0 being a darkness and 100 being a lightness. ^4^ Low = low temperature (12 ± 4.5 °C); Normal = normal temperature (24 ± 3 °C). ^5^ SD = standard deviation.

**Table 6:** The effect of cold stress on malondialdehyde in egg yolk (µM) ^1^.

Item	Temperature				*p*-Value
	Low ^2^		Normal		
	Mean	SD ^3^	Mean	SD	
Day 7	47.47	7.612	41.04	4.098	0.040
Day 14	25.41	3.391	24.17	3.440	0.472
Day 21	51.37	6.537	50.78	4.711	0.818
Day 28	61.85	5.820	65.97	6.866	0.272

^1^ *n* = 10 replicates per treatment. ^2^ Low = low temperature (12 ± 4.5 °C); Normal = normal temperature (24 ± 3 °C). ^3^ SD = standard deviation.

**Table 7:** The effect of cold stress on corticosterone in egg yolk (ng/g) ^1^.

Item	Temperature				*p*-Value
	Low ^2^		Normal		
	Mean	SD ^3^	Mean	SD	
Day 7	257.7	126.29	236.0	87.19	0.740
Day 14	229.0	87.90	198.1	85.83	0.476
Day 21	108.9	56.32	96.6	49.97	0.622
Day 28	125.5	59.12	103.6	55.90	0.445

^1^ *n* = 10 replicates per treatment. ^2^ Low = low temperature (12 ± 4.5 °C); Normal = normal temperature (24 ± 3 °C). ^3^ SD = standard deviation.

**Table 8:** The effect of cold stress on serum biological parameters in serum ^1^.

Item ^2^	Temperature				*p*-Value
	Low ^3^		Normal		
	Mean	SD ^4^	Mean	SD	
TCHO, mg/dL	128.3	18.39	103.5	15.44	0.011
Triglyceride, mg/dL	2487	787.1	1832	567.4	0.047
HDL-CHO, mg/dL	13.20	3.225	14.00	3.300	0.590
GOT, IU/L	116.6	15.53	114.0	11.82	0.679
GPT, IU/L	28.30	1.337	28.50	2.321	0.816
Uric acid, mg/dL	5.130	0.811	4.870	1.200	0.577

^1^ *n* = 10 replicates per treatment. ^2^ TCHO = total cholesterol; HDL-CHO = high density lipoprotein cholesterol; GOT = glutamic oxalacetic transaminase; GPT = glutamic pyruvic transaminase. ^3^ Low = low temperature (12 ± 4.5 °C); Normal = normal temperature (24 ± 3 °C). ^4^ SD = standard deviation.

## Data Availability

The datasets generated or analyzed during the current study are not publicly available but are available from the corresponding author at reasonable request.
